# Connections with Nature and Environmental Behaviors

**DOI:** 10.1371/journal.pone.0127247

**Published:** 2015-05-18

**Authors:** Liuna Geng, Jingke Xu, Lijuan Ye, Wenjun Zhou, Kexin Zhou

**Affiliations:** 1 Department of Psychology, Nanjing University, Nanjing, Jiangsu Province, China; 2 Kuang Yaming Honors School, Nanjing University, Nanjing, Jiangsu Province, China; 3 Nanjing Institute of Environmental Sciences, Ministry of Environmental Protection, Nanjing, Jiangsu Province, China; Saarland University, GERMANY

## Abstract

The influence of environmental attitudes on environmental behaviors has long been discussed. However, few studies have addressed the foundation of such attitudes. In the present study, we explored primitive belief underlying environmental attitudes, i.e., connections with nature, and its relationship with pro-environmental behaviors. Specifically, we used scales, a computerized Implicit Association Test, and a situational simulation experiment to examine both explicit and implicit connections with nature, both deliberate and spontaneous environmental behaviors, and to find correlations between environmental connectedness and environmental behaviors. Results showed that explicit connectedness was positively correlated with deliberate environmental behaviors, while implicit connectedness was positively correlated with spontaneous environmental behaviors. Additionally, explicit and implicit connectedness was independent of each other. In conclusion, the current study confirms the positive role played by connections with nature in promoting environmental behavior, and accordingly suggests means to encourage pro-environmental behavior by enhancing people’s connectedness to nature.

## Introduction

Over the last century, along with rapid growth in technology, we have witnessed increasing environmental problems such as air pollution, water pollution, and global warming. People have gradually acknowledged the substantial influence of human behaviors on the natural world. In environmental psychology, the function of psychosocial variables of pro-environmental behavior, including attitudes, behavioral control, moral norms, intentions, and so forth, has received considerable attention [1].

Research on environmental attitudes, which measures the specific cognitive, affective, and behavioral aspects of attitudes and concerns towards environmental problems, has been fruitful (e.g. [2, 3]). However, few studies have addressed the foundation of attitudes. Set apart from the traditional method, some researchers have attempted to provide theories to explain the development of different attitudes. One example is the New Environmental Paradigm [2, 4], which focuses on the relationship between people and nature, and sees humans as a part of the natural environment. Another example is the Value-Belief-Norm (VBN) model [5]. The model postulates that the value that one place on oneself, other people, or plants and animals provides the foundation for the attitudes one develops about environmental issues, and that different value orientations can lead to different attitudes and activate different behaviors. Following the VBN theory, Schultz and colleagues argue that environmental attitudes and concerns are based on an individual’s primitive belief about the extent to which one is an integral part of the natural environment. They termed this belief as *connections with nature* [6–9].

*Connections with nature* are an important foundation of one’s environmental attitudes [8], and are latent and relatively stable across time and situation. Further, it examines the relationship between human and nature on an individual level. Although the discussion about human–nature relations has a long history in philosophical (e.g. [10]) and sociological literature (e.g. [2, 11]), insufficient attention has been given to this issue in psychology [12]. *Connections with nature* are introduced by Schultz to examine the relationship between an individual and the natural world from a psychological perspective [7–9].

The concept of *connections with nature* has been interpreted in different ways. It was defined by Schultz as “the extent to which an individual includes nature within his/her cognitive representation of self” [7]. Mayer and Frantz [13] viewed connectedness in a different light, as they defined it as an individual’s affective and experiential (rather than cognitive) connection to the natural world. Perrin and Benassi [14] suggested that connectedness with nature is an individual’s beliefs and attitudes about their connection to nature, not mere affective connection. Based on previous studies, we use the term *connectedness with nature* to refer to a factor underlying attitudes towards environmental issues. We define this term as an individual’s feelings (not only emotionally, but also cognitively) regarding connections with nature and belongingness to nature.

### Connectedness with nature and environmental behaviors

As an underlying factor for environmental attitudes, *connections with nature* can be examined both explicitly and implicitly. In this study, we aimed to address the predictive validity of both explicitly measured and implicitly measured *connections with nature* on pro-environmental behavior. Before summarizing the previous research on this topic, it is useful to look back on the psychological mechanism implicated in explicit and implicit measures of attitudes, the relationship between these two measures, and when and why they can predict behaviors.

Explicit and implicit measures involve different psychological processes in behavior determination. Generally, implicit measures, to a greater degree, examine automatic processes [15–17], while explicit measurements largely employ controllable processes that can override automatic processes. Automatic processes are fast, unintentional, involuntary, effortless, and based on an associative network. In contrast, controlled processes are slower, more intentional, under control, effortful, and require heavier cognitive load and higher-order psychological processing [17, 18]. Explicit and implicit measures are related but distinct constructs [19].

Both methods of evaluation have predictive potency for certain contents [20], and the extent to which they can effectively predict behavior depends on moderator variables, such as opportunity to control, motivation to control, and process reliance [17, 21]. Overall, implicit measures will have a higher predictive validity only if individuals rely mostly on automatic processes to guide their behavior and have low opportunity or motivation to control their behavior [17]. On the other hand, explicit measures are supposed to predict behaviors when individuals rely more on controlled processes for behavior determination.

Behaviors vary on a continuum based on the amount of control one exercises [17], and can be generally classified into two types: deliberate and spontaneous behaviors. Measures of deliberate behaviors are mainly self-report testing proxies of behavior, such as behavioral intentions and judgments, whereas measures of spontaneous behaviors are mainly experimental methods [17]. Perugini et al. summarized seven validity patterns (e. g. Double-Dissociation Pattern, Partial-Dissociation Pattern, Double-Addictive Pattern, etc.) to explain the predictive validity of explicit and implicit measures on behaviors. For example, based on the Double-Addictive Pattern, many behaviors are a fairly equal blend of reflective and impulsive processes. In line with Double Dissociation Pattern, controlled and reflective processes have a more significant impact on deliberate behaviors, whereas automatic and impulsive processes affect more deeply spontaneous behaviors [17]. Therefore, it can be inferred that implicit measures should predict spontaneous behaviors, whereas explicit measures should predict deliberate behaviors [21].

In the present study, we measured connection with nature and pro-environmental behavior. Psychologists have developed several explicit measures of connection with nature, such as the Inclusion of Nature in Self (INS) [22], Environmental Identity (EID) [23], Connectedness to Nature (CTN) [13], and Nature Relatedness (NR) [24]. On the other hand, as the Implicit Associations Test (IAT), created by Greenwald, McGhee, and Schwartz [25], is one of the most widely used implicit measures due to its universal and easy applicability [26], Schultz and colleagues have developed several special versions of the IAT to measure connection with nature. These versions of the IAT offer a useful new tool to investigate issues concerning connections with nature [8, 9, 27]. Although there exists a correlation between explicit and implicit measures of connection with nature, it is relatively low [8, 9, 27].

Measurement of environmental behaviors has been challenging for scientists. Current measurement techniques are still mostly based on questionnaire scales. Examples include measuring the environmental behaviors about purchasing, recycling, energy, and transportation [28], and the environmental behaviors of adolescents [29]. Although some evidence shows a modest correlation between the results of these scales and people’s real daily environmental behavior, these scales are not strong predictors of environmental behavior [29]. Researchers have begun to explore experimental methods that simulate real-life situations to examine the degree of spontaneous environmental behaviors (e.g. [30, 31]). In one example, a previous study utilized whether an individual used a free plastic bag as an index of environmental behavior [32]. Similarly, in order to examine the relationship between one’s connections with nature and spontaneous environmental behavior, we used a situational simulation experiment in which the usage of plastic bags was a proxy measure for spontaneous environmental behaviors.

Evidence has accumulated showing a link between connection with nature and pro-environmental behavior. Individuals with higher connectedness are more likely to act in an environmentally friendly manner than are those who feel less connected to nature [33]. It has also been shown that the degree of connectedness with nature can influence the extent to which an individual likes a certain place, thus influencing their willingness and consequent behaviors to protect that place’s pro-environmental environment [34]. Another study, conducted by Howell and his colleagues [35], suggested that people who feel a high degree of connectedness with nature tend to develop more positive life attitudes and engage in more pro-environmental behaviors.

Following the logic in the relationship between explicit and implicit measures and deliberate and spontaneous behaviors, we hypothesized that implicitly measured connections with nature would predict spontaneous pro-environmental behaviors, while explicitly measured connections with nature would predict deliberate pro-environmental behaviors. To our knowledge, few studies have addressed this. In turn, this might be useful for promoting pro-environmental behaviors in real life. In the present study, we incorporated direct scales and the experimental IAT tests to measure connections with nature both explicitly and implicitly, and combine a self-report and a situation simulation experiment to examine pro-environmental behavior.

## Methods

### Participants

One-hundred twenty Chinese students were recruited from Nanjing University. Four participants did not meet the criteria for data analysis used in IAT test. For this reason, they were dropped from the experiment. Moreover, three participants’ statistical data for questionnaires were incomplete. Thus, the final sample included 113 participants (63 male, 50 female; age range: 23–30 years, mean age = 26.54 years, *SD* = 1.81).

### Materials

Participants completed a questionnaire package with two blocks of items: (1) explicit connectedness to nature, and (2) deliberate environmental behaviors. A computerized test was designed to measure implicit connectedness to nature and a situational simulation test examined spontaneous environmental behaviors.

#### Connectedness to Nature Scale (CNS)

The CNS was designed to explicitly measure the degree to which a person feels emotionally connected to nature [13]. Recently, however, it was suggested that the CNS actually measures a cognitive belief about the environment or an environmental attitude [14]. In our study, we follow Schultz [7, 8] and define connections with nature as an individual’s feelings (not only emotionally, but also cognitively) of connection and belongingness to nature. Moreover, past research has shown that CNS can effectively predict environmental behaviors [13, 33].

In our study, we used a Chinese version of the CNS [36]. The CNS contains 14 items regarding the relationship between the individual and nature, for example, “I often feel a sense of oneness with the natural world around me.” Respondents rated each item on a 7-point scale from 1 (*completely disagree*) to 7 (*completely agree*). The scores were totaled, with higher scores indicating that one feels a greater connection to nature.

#### Implicit Association Test (IAT)

A modified Chinese version of the computerized IAT based on that of Schultz and colleagues [8] was created to measure the RT (ms) needed to classify words associated with natural and built environments. The test consisted of seven blocks of 10 trials. The IAT was administered using Inquisit 3.0 software.

In the IAT, participants were timed as they sorted words into four categories using two responses. Two categories were related to the self-concept: Me (*I*, *Me*, *We*, *Mine*, and *Myself*) and Not-Me (*It*, *Other*, *Their*, *They*, and *Them*), and two categories were related to Natural (*Animals*, *Birds*, *Plants*, *Trees*, and *Whales*) and Built (*Building*, *Car*, *City*, *Factory*, and *Street*) environments. The RT needed for each classification was calculated by the computer automatically. Following the introduction, participants were instructed to match words with categories and to decide as fast and accurately as possible. Examples of matching were provided, and all of participants’ questions were answered.

The words were presented in random order within each of the blocks. In order to counter-balance for potential order effects, two versions of the IAT procedure were developed. For participants with an odd identification number, blocks 1, 2, and 5 were considered practice blocks, blocks 3 and 4 were considered “compatible” pairings (e.g., “For a *Me* or a *Nature* word, press the left response key,” and “For a *Not-Me* or a *Built* word, press the right response key”), and blocks 6 and 7 were considered “incompatible” (e.g., “For a *Me* or a *Built* word, press the left response key,” and “For a *Not-Me* or a *Nature* word, press the right response key”). For participants with even identification numbers, blocks 3 and 4 were “incompatible” pairings, and blocks 6 and 7 were “compatible.” According to IAT theory [8], the greater the implicit association one feels between one’s self and nature, the easier and faster one will categorize a compatible pairing. The IAT effect lies in the difference in RTs for the two combinations of Me/Not-Me and Nature/Build words.

We screened outliers for error rate. The average error rate across the 70 trials was 4.2, or 6.0%. Two subjects with a high error rate (error rate > 30%) were dropped. Scores for the IAT were produced using the improved scoring algorithm provided by Greenwald, Nosek and Banaji (2003) [37]. The improved scoring procedure made use of all compatible and incompatible trials. We eliminated trials with latencies over 10,000 ms. Also, we excluded subjects whose latencies of more than 10% of trials were less than 300 ms. As a consequence, we withdrawn two participant’s scores from the final analyses. Then, we replaced each error latency with block mean + 600 ms, and divided the mean reaction time difference between compatible and incompatible trials by the standard deviation to produce a D score. The relative strength of the association between self and nature is indexed by calculating a D-score for each participant [8, 37]. Positive D-scores indicate relatively faster responses when *Me* and *Nature* words are paired (i.e., stronger implicit associations). Negative D-scores indicate relatively faster responses when *Me* and *Built* words are paired (i.e., weaker implicit associations).

#### College Students’ Environmental Behaviors Questionnaire (CSEBQ)

The College Students’ Environmental Behaviors Questionnaire was modified by Shen [38] from the Behavior-based Environmental Attitude scale developed by Kaiser, Oerke, and Bogner [29]. The CSEBQ contains 25 items across seven dimensions: energy conservation, mobility and transportation, waste avoidance, recycling, purchasing/consumerism, food consumerism, and environment supporting (e.g., “I leave the water running when I am washing my clothes,” and “I drive or take a taxi to get around”). The scale was written in the form of a 6-point Likert scale, from 1 (*completely disagree*) to 6 (*completely agree*). Greater total scores were interpreted as reflecting more deliberate environmental behaviors. The scale has been shown to have an acceptable internal consistency (α = 0.76) [38]. The scale also has adequate construct validity, as revealed by the relatively low correlation between different items in the scale but relatively high correlations between each item and the final score [38].

#### Situational simulation experiment

Nestlé wafers of four different flavors (mocha, milk, sesame, and peanut) were given to participants as gifts after they finished the questionnaire and IAT task. Participants were instructed to choose four packets of the same or different flavors, and were then asked whether they needed a plastic bag to pack them up. In the study, the plastic bags were free, but the gifts could easily be held in one hand, which means that the plastic bag was not necessary. When the participant chose to use a plastic bag, this was interpreted as not environmentally friendly behavior. In the present study, using the plastic bag was used as a proxy measure for fewer spontaneous environmental behaviors. The plastic bag used in our study was of an ordinary type, similar to those commonly used in daily shopping. It was transparent and had no picture on it. The bag’s average appearance allowed is to rule out other possible factors possibly influencing one’s decision. Furthermore, by asking participants to choose wafer flavors, we prevented them from focusing on the plastic bag itself, thus raising the likelihood of spontaneous behavior.

Using a plastic bag was marked as a score of “0,” while not using one was marked as “1.” Higher scores indicated a higher degree of spontaneous environmental behavior.

### Procedure

All participants were tested individually in a private room. First, participants provided written consent to participate in the study. Each participant completed the questionnaire, the IAT, and the situational simulation test. Half of the participants finished the questionnaire first and then performed the IAT, while the other half completed the IAT first and then the questionnaire. After participants completed the questionnaire and the IAT, the situational simulation experiment was conducted. Finally, participants were thanked and debriefed. This study was approved by Institutional Review Board of Nanjing University.

## Results

Data were obtained from 113 participants. Statistical analyses were conducted with SPSS 19.0. The data were screened according to the principle of 3 standard deviations above or below the mean scores, and we found no outliers in the questionnaires. The internal consistencies of the scales used were within an acceptable range. Cronbach’s alpha for the CNS was 0.71, and the mean score was 78.98 (*SD* = 8.02). Alpha reliability for the CSEBQ was 0.64. The mean score of the CSEBQ scale was 105.37 (*SD* = 10.05). The mean IAT D-scores ranged from -1.29 to 1.32 (*M* = 0.03, *SD* = 0.48). The internal reliability, calculated by correlating the two IAT subscales (i. e. Block 6—Block 3, with Block 7- Block 4), was. 80 (*p*<.01). In the plastic bag situational simulation test, 40 participants refused the plastic bag (35.4%) and 73 participants used one (64.6%). There was no significant effect of gender or education level on any scale or test.

Correlation coefficients were computed to evaluate bivariate relationships among the four variables of interest: CNS, D-scores, Bag usage, and CSEBQ. The results are shown in Table 1. As seen in the table, no significant correlation was found between CNS and D-scores (*r* = 0.04, *p* = 0.66). This result indicates that explicit connectedness to nature and implicit connectedness to nature are independent of each other. Interestingly, CSEBQ was significantly correlated with CNS (*r* = 0.39, *p* < 0.001), and with Bag usage (*r* = -0.23, *p* = 0.02), but not with D-scores (*r* = -0.14, *p* = 0.13). In addition, there was no significant correlation between Bag usage and CNS (*r* = -0.12, *p* = 0.22), but the correlation between Bag usage and D-scores was strong and significant (*r* = 0.56, *p* < 0.001). Therefore, we can infer that deliberate environmental behaviors are not associated with implicit connectedness to nature, and spontaneous environmental behaviors are not associated with explicit connectedness to nature.

**Table 1:** Correlations between CNS, D-scores, CSEBQ, and Bag usage.

	CNS	D-score	CSEBQ	Bag usage
**CNS**	1.00			
**D-scores of IAT**	0.04	1.00		
**CSEBQ**	0.39**	-0.14	1.00	
**Bag usage**	-0.12	0.56**	-0.23*	1.00

CNS: Connectedness to Nature Scale; IAT: Implicit Association Test; CSEBQ: College Students’ Environmental Behaviors Questionnaire.

*p < 0.05,

**p < 0.01.

Linear regression using the enter method was employed to test if CNS and D-scores could be used to predict CSEBQ, i.e., deliberate environmental behaviors. Results revealed that the CNS and D-score combined were significant predictors of deliberate environmental behaviors measured by the CSEBQ (*R* ^*2*^ = 0.17, *F* = 11.59, *p* < 0.001). However, only CNS made a significant unique contribution (*β* = 0.39, *t* = 4.52, *p* < 0.001). D-scores did not significantly predict CSEBQ (*t* = -1.85, *p* = 0.07). This suggests that explicit connection with nature is a strong predictor of deliberate environmental behaviors.

Based on the findings above, we employed a binary logistic regression analysis to explore the factors that predict spontaneous behaviors. Bag usage was defined as the criterion variable, and D-score and CNS were set as predictor variables. D-scores and CNS were significant combined predictors of spontaneous environmental behaviors as measured by bag usage (χ^2^ = 9.35, *p* = 0.002). However, D-score made the only significant unique contribution to spontaneous environmental behaviors (*Wald* χ^2^ = 23.93, *p <* 0.001), and CNS scores did not significantly predict bag usage (*Wald* χ^2^ = 2.53, *p* = 0.11). The odds ratio (OR) was 3.8 for D-scores in the analysis equation, indicating that greater D-scores were associated with a greater likelihood of not using the bag. In other words, the level of one’s implicit connections with nature correlated positively with their rate of spontaneous environmental behavior. The results support our hypothesis that implicit connections with nature can predict spontaneous behaviors.

## Discussion

The results demonstrate that connections with nature are an effective predictor of pro-environmental behavior, which corresponds with previous research [13, 33, 35]. Moreover, our study indicates that explicit connections with nature predict deliberate environmental behaviors, while implicit connections with nature predict spontaneous environmental behaviors.

The results showed a very weak correlation (*r* = 0.04) between explicit and implicit connections with nature. This is inconsistent with previous studies [8, 9, 27] indicating that the implicit IAT measure is positively correlated with explicit measures of inclusion in nature, despite the fact that the correlations reported were small. It is noteworthy that Schultz and colleagues used the Inclusion of Nature in Self (INS) scale, which is a single-item graphical measure, as the explicit measure. This scale is different from the CNS, which was utilized in the present study. Although some researchers have questioned whether CNS is affective, arguing that it instead measures cognitive beliefs [14], it has been acknowledged that CNS measures more of an emotional connection with nature [13]. The INS, however, focuses on cognitions. This might explain why the CNS does not correlate with the IAT-nature, while the INS does. Further, relationships between implicit and explicit measures are determined by many moderator factors. For example, Nosek [39] points out that *self-representation*, *evaluation strength*, *dimensionality*, and *distinctiveness* can each moderate the implicit–explicit relationship. Therefore, more research is needed to explore the relationship between explicit and implicit connections with nature.

The finding that IAT-nature predicts spontaneous behaviors whereas CNS predicts deliberate behaviors provides empirical support for the different types of psychological processing involved in explicit and implicit measures. Moreover, our findings correspond with the double dissociation pattern. This pattern has already received empirical support from a number of studies, and provides a useful theoretical view for examining the mental processes in behavior determination (e.g. [40, 41]). For example, Asendorpf and colleagues [40] showed that a shyness IAT uniquely predicted spontaneous but not deliberate behavior, whereas self-reports uniquely predicted deliberate but not spontaneous behavior. This can also be explained by the correspondence principle [42], and the strength of relation between a construct measure and a behavioral measure relies on the degree of similarity in their features [21]. In our study, the CNS and the CSEBQ both assessed largely controlled processes, whereas the IAT-nature and bag choice task both assessed mainly automatic processes. Besides, from the perspective of methodology, the CNS and the CSEBQ are both formatted and delivered in a similar way and measured with Likert scales. Whereas the IAT has an additional context category used of the built environment that is of a lesser focus in the explicit task. Hence, the CNS was expected to predict the CSEBQ while the IAT-nature predicts bag usage.

Interestingly, the result revealed that D-score made a marginally significant contribution to CSEBQ scores (*t* = -1.85, *p* = 0.07), and the p-value of CNS scores for predicting Bag choice was also not far from significance (*p* = .11). These results can be explained in terms of the recent finding that measurement outcomes reflect both automatic and controlled processes (e.g. [43, 44]). Following this perspective, we can infer that primarily influenced by automatic processes, implicit measures are also affected by controlled processes. The same holds for explicit measures. Hence, to a certain degree implicitly measured connectedness can have an impact on deliberate behaviors while explicitly measured connectedness can make contribution to spontaneous behaviors. In addition, the negative correlation between IAT D-scores and CSEBQ was unexpected, though not significant. The following reason might explain this outcome. The IAT procedure in the present study is composed with only 70 trials as referred to Schultz et al. rather than a regular IAT which usually includes 200 trials [8]. The number of trials might be too small for measuring implicit connections with nature. For future study, the IAT procedure should be modified to employ the regular version of IAT. Other methodological reasons need to be explored further.

### Implications

There was a strong correlation between implicit measures of environmental connectedness and spontaneous pro-environmental behavior (*r* = 0.56). However, the predictive validity of explicit and implicit measures depends upon moderator variables, as it varies under different dispositional, situational, and behavioral circumstances [17, 41]. A number of empirical studies have supported this argument [17, 20]. For instance, research has found that motivations regarding impression management and self-presentation can undermine the predictive validity of explicit measures in socially sensitive domains, but have a much weaker effect on implicit measures [20, 21]. Hence, in our study, it might be the case that in order to present oneself favorably, participants were more likely to report higher connectedness, which led to reduced validity of explicit measures in predicting spontaneous pro-environmental behavior. In contrast, the predictive validity of implicit measures remains high because of its independence from social sensitivity. As another example of this phenomenon, Marsh, Johnson, and Scott-Sheldon [45] found that condom use varies across situations, and that an attitudinal IAT correlates with condom use in a casual relationship but not with condom use in a steady relationship. Similarly, in our study, we can infer that participants’ accepting a plastic bag to take the gift is more of a casual behavior rather than it is a stable behavior, like using plastic bags in everyday shopping. As a result, the moderators of the predictive validity of explicit and implicit measures require further investigation in the field of environmental psychology.

The study inspired us to ponder on how to ignite people’s passion to protect nature. In the long term, it would be wise to cultivate people’s connection with nature, promote the emotional and cognitive tie between humans and the natural world, and increase people’s feeling of being one with nature. Previous research has also demonstrated that connectedness promotes pro-environmental behaviors [13, 33]. As connection with nature is a primitive belief that is not prone to changes in a short time, it is essential to restore one’s connection with nature long-term. It would be effective to spend more time in the natural world and have direct contact with nature (e.g., camping in the park, hiking in the mountains) to increase connectedness. In doing so, individuals form a bond with nature, thus maintaining a pro-environmental lifestyle that then activates pro-environmental behaviors [27, 33].

### Limitations and future directions

Although this study reveals innovative and interesting findings regarding the relation between connection with nature and pro-environmental behavior, we would be remiss if we did not note some limitations of the study. First, the relationship between connection with nature and environmental concerns or attitudes has not been fully investigated. Despite our claim that connection with nature is an important foundational factor of environmental attitudes that can predict individuals’ pro-environmental behavior, future studies must yet develop a theoretical model for the relationship between connection with nature and environmental concerns or attitudes. Second, the structure of the plastic bag test to measure spontaneous environmental behavior is binary, not continuous. The measurement might thus be oversimplified, necessitating the development of a more elaborate tool in future studies.

We conclude that our study provides evidence for the correlation between connection with nature and environmental behaviors, and further suggests that explicit connection with nature can predict deliberate pro-environmental behavior, whereas implicit connections with nature can predict spontaneous pro-environmental behavior. Thus, it is implicit connection with nature that can genuinely predict pro-environmental behaviors in real-life situations.
